# A Test Data Compression Scheme Based on Irrational Numbers Stored Coding

**DOI:** 10.1155/2014/982728

**Published:** 2014-08-28

**Authors:** Hai-feng Wu, Yu-sheng Cheng, Wen-fa Zhan, Yi-fei Cheng, Qiong Wu, Shi-juan Zhu

**Affiliations:** ^1^School of Computer and Information, Anqing Normal University, Anqing 246011, China; ^2^Department of Science Research, Anqing Normal University, Anqing 246011, China

## Abstract

Test question has already become an important factor to restrict the development of integrated circuit industry. A new test data compression scheme, namely irrational numbers stored (INS), is presented. To achieve the goal of compress test data efficiently, test data is converted into floating-point numbers, stored in the form of irrational numbers. The algorithm of converting floating-point number to irrational number precisely is given. Experimental results for some ISCAS 89 benchmarks show that the compression effect of proposed scheme is better than the coding methods such as FDR, AARLC, INDC, FAVLC, and VRL.

## 1. Introduction

According to Moore's law, the integration level of the microchips doubles every 18 to 24 months. As a result, the volume of test data increased dramatically; the test costs become higher; the contradictions between test efficiency and test quality are sharpening. The test data compression technology comes into being to solve the above problems.

Coding compression technology has been widely used because it has such advantages as simple encoding and decompression structure which is independent of the tested chip. A certain encoding scheme is used to encode the original test set. The length of the encoded data is less than the original test set, so as to reduce the test data.

The test set is divided into the sequences of specific law, which can be replaced with new codeword generated by some kinds of coding method. According to the change rule of the length from the original sequence to the codeword, coding methods can be divided into four categories. The first category is fixed-to-fixed coding method, such as dictionary code [1]. The second category is fixed-to-variable coding method, such as Huffman code [2], 9C code [3]. The third category is variable-to-fixed encoding method, such as run-length code [4]. The fourth category is variable-to-variable coding method, such as Golomb code [5], FDR code [6], EFDR code [7], and alternation and run-length code [8].

Among them, the compression method of the first category is the most simple one, but it has the lowest compression efficiency. The compression efficiency of the fourth category is high, but its hardware overhead is larger. The second and third categories have good applicability, which are between the first and fourth categories in terms of compression method, compression efficiency, hardware overhead, and so forth.

It is a new original method to use irrational number to compress test data. It is creative. A scheme of test data compression based on irrational number dictionary code [9] is presented. The encoding rule of this scheme is simple, and the do not-care bit need not be assigned, but it takes extra storage space to store the data dictionary additionally.

A new test data compression scheme based on irrational numbers stored (INS) is presented. It is a fixed-to-variable coding method, which has simple encoding rule and can obtain good compression effect. In this scheme, the test set is converted into floating-point numbers firstly, and then the floating-point numbers are converted into irrational numbers in form of $\sqrt[{r}]{x}$ (where the numbers *x* and *r* are integers) by the successive approximation method. So the storage of the test set can be translated into the storage of radicand (integer) and root number (integer). The minimum of corresponding radicand and root number can be found with faster convergence speed. Better compression effect can be obtained when using the INS coding scheme.

The organization of the paper is as follows.  Section 2 explains the algorithm of the proposed scheme and gives an example.  Section 3 proves the feasibility of the algorithm of the proposed scheme theoretically. The structure of decompressor is presented in Section 4.  Section 5 reports the experimental results and analyzes the compression ratio theoretically. Finally Section 6 concludes the paper.

## 2. INS Coding

In this section, the algorithm of INS coding is described firstly. Then, the flowchart of the scheme is given. Lastly, an example is provided.

### 2.1. Encoding Rule

The concrete steps of INS coding are as follows.

1. At first, generate the determinate complete test set named *T* by automatic test pattern generation tools.
2. Then cascade the entire test vector, and connect the head of a vector to the tail of previous vector, remembered as *S*.
3. Take the first *N* bits of the test set, and then convert them into a hexadecimal number every four bits a group according to the rules shown in Table 1. Add a decimal point after the first digit, and a hexadecimal floating-point number named *f* is formed.
4. Calculate the value of *x* and *r* in the formula $\sqrt[{r}]{x}=f$, by dichotomy to successive approximate *f*. In this scheme, if the first several bits of *f* and the root of $\sqrt[{r}]{x}$ are exactly the same, that is to say, the two are approximately equal to, then consider the following. (I) Calculate *f* ^2^ first; set bot = ⌊*f* ^2^⌋, top = bot + 1, *r* = 2. (II) Calculate $\sqrt[{r}]{\text{bot}}$, if its value is equal to *f*, note *x* = bot, *r*, and then go to step (5). Otherwise, calculate $\sqrt[{r}]{\text{top}}$, if its value is equal to *f*, note *x* = top, *r*, and then go to step (5). (III) Consider$\text{bot}=\left\lfloor \text{bot}*\sqrt[{r}]{\text{bot}}\right\rfloor ,\text{top}=\left\lceil \text{top}*\sqrt[{r}]{\text{top}}\right\rceil$, and *r* = *r* + 1. (IV) Set mid = ⌊(bot + top)/2⌋, calculate $\sqrt[{r}]{\text{mid}}$, if its value is equal to *f*, note *x* = mid, *r*, and then go to step (5). If its value is greater than *f*, top = mid − 1. If its value is less than *f*, bot = mid + 1. Repeat step (IV) until bot > top, and then go to step (V). (V) If top is less than mid, set bot = top, top = mid. Otherwise, set top = bot, bot = mid. Repeat step (4) until $\sqrt[{r}]{x}=f$ is valid and go to step (5). In this step, the median (mid) is always integer in operation process. That can reduce the computation complexity, save the running time, and accelerate the operation process.
5. Encode *x* and *r* in the form of CEBM [10]. Remove the first *N* bits of *S* and repeat steps (3) and (4), until *S* is empty.

**Figure 1:**
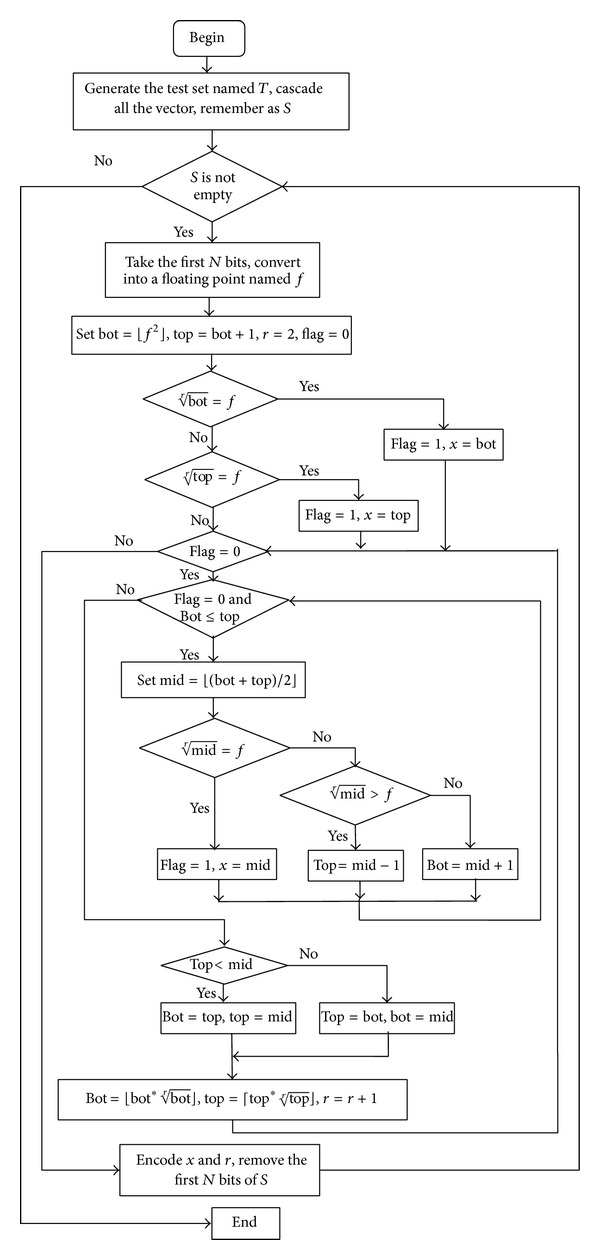
Flowchart of INS coding.

Figure 1 shows the detailed flowchart of the scheme.

CEBM is shown in Table 2. Because *x* ≥ 2, *r* ≥ 2, the run-length starts from 2. The first column is the length of runs and the second column is the number of group. The third column is odd bits of the codeword and the fourth column is even bits. The last column is the corresponding codeword. As can be seen, CEBM has the following characteristics. (1) The length of the odd bits and the even bits is equal to every codeword. (2) The odd bits show the run-length. Add data “1” before the odd bits; the new odd bits are just the corresponding binary number of the run-length. The even bits show the end of the codeword. The codeword continues if the even bit is 0 and finishes if it is 1. (3) The length of codeword increases by two bits from group *A*_*i*_ to group *A*_*i*+1_; both the odd bits and the even bits increase one bit. (4) Group *A*_*i*_ contains 2^*i*^ elements. (5) The corresponding relationship between the run-length *L* and group *A*_*i*_ is like *i* = ⌈Log_2_(*L* + 1) − 1⌉.

For an example, the codeword of 9 is 000011. The odd bits are 001 and the even bits are 001. The even bit needs to be monitored when decoding. If the even bit is 0, it means that codeword continues. If the even bit is 1, it means that codeword finishes. Add data “1” before the odd bits (001); we can get the data 1001, whose corresponding decimal value is 9. CEBM is widely used because of decoding simply and small hardware overhead.

Through the analysis, the scheme has the following three characteristics. (1) The root number is calculated from 2 and increases successively. It can guarantee that the radicand and root number found are minimum. (2) The lower bound and upper bound of the radicand's interval positioned are relatively accurate. At the same time, the binary search method can reduce the time complexity. (3) In the binary search, the medians of the operation process are all integers. It can reduce the computation complexity, save the running time, and accelerate the process of operation.

### 2.2. Encoding Example

An example is provided to make the scheme clear. Without loss of generality, set the original test set *T* = {00011010, 11101000, 10011111, 10011001, 01011010, 11010011, 11101001, …}. Cascade all the test vector, and then divide it into sequences of 48 bits. The data flow is 000110101110100010011111100110010101101011010011 11101001… . Its first 48 bits can convert into a hexadecimal floating-point *f* = 1.AE89F995AD3. (I) Calculate *f* ^2^ = 2.D413CCCFE7551FCA6F09E9, bot = ⌊*f* ^2^⌋ = 2, top = bot + 1 = 3, *r* = 2. (II) Calculate $\sqrt[{r}]{\text{top}}=\sqrt[{2}]{3}=1$.BB67AE8584C, and go to step (III) because its value is not equal to *f*. (III) Consider $\text{bot}=\lfloor \text{bot}*\sqrt[{r}]{\text{bot}}\rfloor =\lfloor 2*\sqrt[{2}]{2}\rfloor =\lfloor 2.\text{D}413\text{CCCFE}76\rfloor =2$, $\text{top}=\lceil \text{top}*\sqrt[{r}]{\text{top}}\rceil =\lceil 3*\sqrt[{2}]{3}\rceil =\lceil 5.32370\text{B}908\text{E}4\rceil =6$, *r* = *r* + 1 = 3. (IV) Set mid = ⌊(bot + top)/2⌋, and calculate $\sqrt[{r}]{\text{mid}}$. If its value is equal to *f*, note *x* = mid, *r*, and go to step of encoding. If its value is greater than *f*, set top = mid − 1. If its value is less than *f*, set bot = mid + 1. Repeat step (IV) until bot > top  (bot = 5, top = 4), and go to step (V). (V) If top is less than mid, set bot = top, top = mid. Otherwise, set top = bot, bot = mid. Then bot = 4, top = 5. Repeat steps (III), (IV), and (V) and we can get the results *r* = 4, *x* = 8, which make $\sqrt[{4}]{8}=1$.AE89F995AD3. So the storage of the first 48 bits of the data flow 00011010111010001001111110011001010110101101001111101001… can be converted into the storage of the radicand (8) and the root number (4). The CEBM of 8 and 4 is 000001 0001, a total of 10 bits, reducing 38 bits.

## 3. Theoretical Analysis

In this section, the theoretical analysis of the algorithm is given.

For any floating-point number *f*, there exist positive integers *x*, *r*, which make the first several bits of *f* and the root of $\sqrt[{r}]{x}$ to be exactly the same; that is to say, the two are approximately equal.

The process of proof is as follows.

From the encoding rule of Section 2.1, *f* is positioned in the interval [$\sqrt[{r}]{\text{bot}}$, $\sqrt[{r}]{\text{top}}$] through binary search each time in the process of successive approximation, where

(1) $$\begin{array}{c} \text{bot}=\lfloor f^{r}\rfloor ,\\ \text{top}=\text{bot}+1=\lfloor f^{r}\rfloor +1. \end{array}$$

Therefore, we only need to prove that $\sqrt[{r}]{\text{bot}}$ is infinitely close to $\sqrt[{r}]{\text{top}}$ when *r* → *∞*. That is to prove

(2) lim⁡r→∞⌊fr⌋+1r⌊fr⌋r=1lim⁡r→∞⌊fr⌋+1r⌊fr⌋r=lim⁡r→∞eln⁡⁡((⌊fr⌋+1r)/⌊fr⌋r)=lim⁡r→∞eln⁡((⌊fr⌋+1)/⌊fr⌋)1/r=lim⁡r→∞e(1/r)ln⁡((⌊fr⌋+1)/⌊fr⌋)lim⁡r→∞1rln⁡(⌊fr⌋+1⌊fr⌋)=lim⁡r→∞ln⁡(1+1/⌊fr⌋)r=0lim⁡r→∞⌊fr⌋+1r⌊fr⌋r=lim⁡r→∞e0=1.

That is to say, we can always find positive integers *x* and *r* to make $\sqrt[{r}]{x}$ and *f* to be approximately equal when *r* is increasing.

## 4. Structure of Decompressor

In this section, the structure and basic operation principle of the decompressor for the INS coding scheme are illustrated.

**Figure 2:**
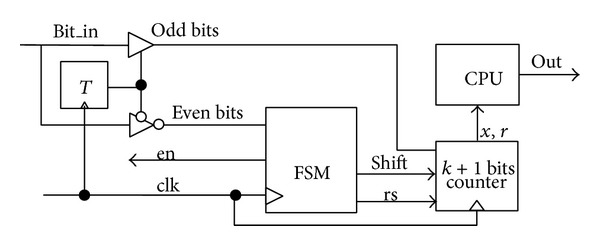
Structure of decompressor.

Figure 2 shows the structure of decompressor, which contains a finite state machine, a T flip-flop, a special *k* + 1 bits counter and some combinational logic, and the CPU module on SoC chip.

This special *k* + 1 bits counter is unique. Set its lowest bit as 1; move this bit to high with other data when the data needed to be decoded is moved to the counter. The function can be realized by a combination of some simple circuit, and the hardware overhead will not increase significantly. In the *k* + 1 bits counter, the value of *k* is decided by the maximum of the radicand and the boot number (named as *L*_max⁡_) in compression result, *k* = ⌈log_2_(*L*_max⁡_ + 1)⌉−1.

The root operation can be realized by the CPU module on SoC chip as long as the floating-point processing unit ×87 FPU integrated in CPU. ×87 FPU has its own instruction system, including the commonly used instruction types: floating-point move instructions, floating-point arithmetic operation instructions, floating-point transcendental function instructions, floating-point comparison instructions, and FPU control instructions [11]. Floating-point transcendental function instructions can realize exponent operation (F2XM1 instruction) and logarithmic operation (FYL2X instruction). $\sqrt[{r}]{x}$ can be converted into 2^(log_2_*x*)/*r*^, which can be calculated by exponent operation instruction and logarithmic operation instruction.

Decompressor works as follows. (1) First, the FSM makes an enable signal named* en* into high level, and the encoded data named* bit_in* is split into two parts: odd bits and even bits by the position of data. That is implemented by the T flip-flop. Odd bits are directly shifted into the *k* + 1 bits counter, and even bits are shifted into FSM. (2) Then, FSM repeats to read the encoded data until the value of even bit equals 1. Meanwhile, the odd bit is shifted into the *k* + 1 bits counter. (3) The data (named *x*) of the *k* + 1 bits counter is shifted into CPU after all odd bits of data are received in the special *k* + 1 bits counter. Repeat step (2); the data of the *k* + 1 bits counter (named *r*) is shifted into CPU. (4) $\sqrt[{r}]{x}$ is calculated by floating-point processing unit ×87 FPU in CPU, and the first *N* bits of its binary form are outputted.

## 5. Experimental Results

In this section, the effectiveness of the INS coding scheme is verified by using experimental results.

INS coding is applied to the MinTest test sets of the largest ISCAS 89 benchmark circuits. The experimental results are shown in Table 3. The first column shows the circuit name, the second column shows the total number of the data in the original test sets, the third column shows the total number of the compressed data, and the fourth column shows the compression ratio.

In order to verify the validity of this scheme, compare with the similar algorithm, as shown in Table 4. Among them, the first column shows the circuit name, the second to sixth columns, respectively, show the compression effect of FDR code [6], INDC code [9], AARLC code [8], FAVLC code [12], and VRL code [13], and the seventh column shows compression effect of the scheme presented. Data from Table 3 shows this scheme besides lower compression ratio in the fifth circuit, and the rest of the circuit has higher compression ratio. And this scheme has a good compression effect on the whole. The average compression ratio reaches 65.79%, which is 20.46%, 9.42%, 7.36%, 5.36%, and 1.85% higher than those of FDR, RLR, AARLC, FAVLC, and VRL.

In order to further demonstrate the effectiveness, the results of proposed scheme are compared with other schemes under the same circumstances by the hard fault set of some ISCAS 89 benchmark circuits. The results as shown in Table 5. As can be seen from Table 4, the overall effect of proposed scheme is better. The average compression ratio of the proposed scheme is 3.87%, 3.82%, and 3.31% higher than those of FDR, AR [14], and ARL [15]. These data show the effectiveness of INS Code.

The experimental results indicate the following. (1) Regardless of the fact that the determined bit of the test data is 0 or 1, it has a little effect on compression ratio in the process of finding the irrational number. (2) Do not-care bit can accelerate the search speed of irrational number, reduce the value of irrational number used to store the test data, and improve the compression ratio.

The influence of the do not-care bit probability on compression is explored. The length of the test data fragment is set to be *K*, and the probability of do not-care bit is set to be *ρ*. Their relationship can be expressed in the following formula:

(3) $$\begin{array}{c} \sqrt[{r}]{x}-f_{i}<10^{-(K/4)+1},\;i=1,2,\ldots ,2^{K\cdot \rho }, \end{array}$$

where, *x*, *r*, and *K* are integers and *f*_*i*_ is a floating-point number.

The compression gain can be calculated by the following formula:

(4) $$\begin{array}{c} \beta =\frac{K}{\left(1+\text{lo}{\text{g}}_{2}\left(x+1\right)+\text{lo}{\text{g}}_{2}\left(r+1\right)+\text{lo}{\text{g}}_{2}\left(K+1\right)\right)}. \end{array}$$

**Figure 3:**
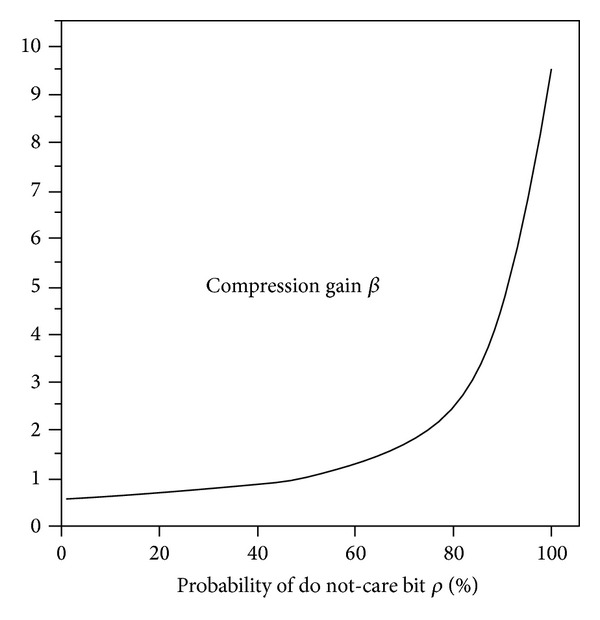
Relationship between *β* and *ρ*.

By analyzing the experimental data, the relationship between the compression gain *β* and the probability of not-care bit *ρ* can be concluded as shown in Figure 3.

From Figure 3, as can be seen, the proposed compression scheme can obtain better compression effect in a higher probability of do not-care bit.

## 6. Conclusion

INS coding is presented, which uses the floating-point number unfolded by irrational number to store the test set. It is creative. Using the successive approximation method can accelerate the convergence speed in the process of searching the irrational number. The experimental results show that the compression effect of the scheme is better. It provides a new choice to solve the problem of test data compression.

## Figures and Tables

**Table 1:** Test data transformation rules.

Test data	Hexadecimal number	Test data	Hexadecimal number	Test data	Hexadecimal number	Test data	Hexadecimal number
0000	0	0100	4	1000	8	1100	C
0001	1	0101	5	1001	9	1101	D
0010	2	0110	6	1010	A	1110	E
0011	3	0111	7	1011	B	1111	F

**Table 2:** CEBM.

Run-length	Group	Odd bits	Even bits	Codeword
2	*A*_1_	0	1	01
3		1	1	11

4	*A*_2_	00	01	00 01
5		01	01	00 11
6		10	01	10 01
7		11	01	10 11

8	*A*_3_	000	001	00 00 01
9		001	001	00 00 11
⋮		⋮	⋮	⋮
14		110	001	10 10 01
15		111	001	10 10 11

⋮	⋮	⋮	⋮	⋮

**Table 3:** Compression effect of scheme presented.

Circuit	Size of TD bit	Scheme presented	
		Size/bit	*α*%
S5378	23754	9868	58.46
S9234	39273	16157	58.86
S13207	165200	24998	84.87
S15850	76986	22555	70.70
S38417	164736	81611	50.46
S38584	199104	56981	71.38

Avg.			65.79

**Table 4:** Comparison with other algorithms.

Circuit	FDR	AARLC	INDC	FAVLC	VRL	Scheme presented
S5378	48.02	45.12	45.05	52.15	52.14	**58.46**
S9234	43.59	42.79	47.08	45.82	50.17	**58.86**
S13207	81.30	80.43	**85.27**	81.58	83.29	84.87
S15850	66.22	65.13	67.24	67.70	69.78	**70.70**
S38417	43.26	56.52	47.61	43.06	**62.84**	50.46
S38584	60.91	60.57	66.02	**72.29**	65.42	71.38

Avg.	45.33	58.43	59.71	60.43	63.94	**65.79**

**Table 5:** Comparison with other algorithms (the hard fault set).

Circuit	Size of TD bit	FDR	AR	ARL	Scheme presented	
					Size/bit	α%
S5378	5992	1110	1160	1091	**710**	88.15
S9234	73112	22038	21222	20429	**11982**	83.61
S13207	220500	14080	15534	15061	**7500**	96.60
S15850	163748	14370	13286	**12861**	15183	90.73
S38417	2201472	103760	94682	**90397**	103779	95.29
S38584	456768	13840	15696	15083	**12551**	97.25
